# Research on the Release of Dangerous Compounds from the BTEX and PAHs Groups in Industrial Casting Conditions

**DOI:** 10.3390/ma14102581

**Published:** 2021-05-16

**Authors:** Mariusz Holtzer, Rafał Dańko, Sylwester Piasny, Michał Kubecki, Dariusz Drożyński, Agnieszka Roczniak, Mateusz Skrzyński, Angelika Kmita

**Affiliations:** 1 Faculty of Foundry Engineering, AGH University of Science and Technology, Mickiewicza 30 Str., 30-059 Krakow, Poland; holtzer@agh.edu.pl (M.H.); dd@agh.edu.pl (D.D.); arocznia@agh.edu.pl (A.R.); mskrzyns@agh.edu.pl (M.S.); 2 HARDKOP Sp. z. o. o., Harcerska 12 Str., 32-540 Trzebinia, Poland; sylwester.piasny@hardkop.pl; 3 Department of Analytical Chemistry, Łukasiewicz Research Network—Institute for Ferrous Metallurgy, K. Miarki Str., 12-14, 44-100 Gliwice, Poland; michal.kubecki@imz.pl; 4 Academic Centre for Materials and Nanotechnology, AGH University of Science and Technology, Mickiewicza 30 Str., 30-059 Krakow, Poland

**Keywords:** environmental protection, foundry industry, hazardous pollutants, emission, BTEX, PAHs

## Abstract

The assessment of the harmfulness of moulding and core sands is mainly based on investigations of compositions of gases emitted by liquid casting alloys during the mould pouring. The results of investigations of moulding sands obtained under industrial conditions are presented in this paper. A unique research stand was designed and built for this aim. It allowed us to determine emissions of gases at individual stages of casting a mass up to 50 kg. This approach enables simulation of foundry conditions. Moulding sands bound by organic binders (phenol-formaldehyde; furan), inorganic binders and green sand, were subjected to investigations. The composition of gases that evolved during the individual stages, pouring, cooling and knocking out, was tested each time, and the contents of Polycyclic Aromatic Hydrocarbons (PAHs) and benzene, toluene, ethylbenzene, and xylenes (BETX) were analysed. Investigations indicated that the emission of gases from sands with inorganic binders is negligible when compared with the emission of gases from sands with organic binders. The emission of gases from green sand is placed in the middle of the scale. As an example: the sand with furan resin emitted 84 mg of BTEX (in recalculation for 1 kg of sand) while from sands with inorganic binders there was a maximum of 2.2 mg (for 1 kg of sand). In the case of sands with inorganic binders, MI and MC sands indicated comparable and very low emissions of gases from the PAHs group, at the level of 0.018 mg and 0.019 mg for 1 kg of sand, respectively. The higher emission of PAHs from MG sand is the result of its different way of hardening (a binder was of an organic character) than of sands MI and MC.

## 1. Introduction

In foundry plants, metal casting can be done with various methods. One of the most common methods preferred around the world is sand casting. Moulding sands, in which the castings are produced, can be bound by organic binders (e.g., furan, phenol-formaldehyde resins), inorganic binders (water glass, aluminosilicates) or by bentonite. Under the influence of the high temperatures of liquid metal, there is a hazard of emitting dangerous substances from a mould: benzene, toluene, ethylbenzene, xylenes (BTEX) and Polycyclic Aromatic Hydrocarbons (PAHs) groups [1]. The gas evolution performance of the mould is a very important index, which is directly related to the quality of casting [2,3,4]. The main reason for testing the emission of compounds from the BTEX or PAHs group is that some of these compounds show carcinogenic and/or mutagenic properties. Several PAHs show genotoxic, mutagenic and carcinogenic properties, e.g., benzo(a)pyrene that the body can metabolize to carcinogenic form. PAH derivatives with incorporated nitrogen atoms (NPAH) are characterized by several hundred greater carcinogenic accents. Therefore, it is important to reduce PAH exposure to improve public health [5].

A range of exposure standards for BTEX are in use around the world. Benzene is now a regulated pollutant in the European Union, and the United States has introduced regulations for industrial emissions monitoring. The World Health Organization (WHO) and International Agency for Research on Cancer (IARC) classify benzene as a group one carcinogen. Prolonged exposure to high concentrations of benzene causes leukaemia and impacts red and white blood cells. Hence, monitoring BTEX is also extremely important from the point of view of environmental protection and human health [6].

The gas evolution performance of the mould is a very important index, which is directly related to the quality of casting [2,3,4].

The kind of substances which are formed when sand moulds are poured with liquid alloys depends mainly on the atmosphere inside the mould cavity [7,8,9]. This atmosphere depends on the applied binder, additions to sand, and liquid metal temperature [4,8,9,10,11,12,13].

In the initial phase, the mould cavity is filled with air, which means that there is an oxidising atmosphere. While the mould is filling with liquid metal, the air is pushed out by riser heads and through venting holes transported into the depth of the mould. The basic element of the carbon chemical structure is the carbon skeleton, with which hydrogen is the most often bound. In case of binders of organic origin (e.g., resins, additives containing carbon) under an influence of high temperatures, chains containing carbon are subjected to sudden disintegration with emissions of hydrogen and/or nitrogen (the so-called dehydrogenation), which causes the atmosphere inside the mould cavity to become the reducing one [14,15,16].

Reactions occurring in the mould can then be compared to the so-called “flash pyrolysis” [3,4,10,13,17]. As time goes by, the heat front shifts from the casting deep into the mould (this process can be called “slow pyrolysis”), causing a further destruction of the polymer binder. This process continues until the mould-casting system reaches the binder decomposition temperature (400–600 °C). The part of sand in direct contact with the air becomes burned, and due to that, CO and CO_2_ are formed [18,19,20,21,22,23]. 

In the case of green sand (silica sand + bentonite + water) during the casting process and the subsequent gasification of moisture contained in bentonite bonded sand, the mould atmosphere is saturated with highly reactive concentrations of oxygen and hydrogen, creating an extremely reactive oxidising atmosphere [18,24,25,26]. In the atmosphere rich of oxygen and hydrogen, oxygen easily reacts with carbon contained in cast iron or the sand additives containing carbon, and in result, CO and CO_2_ are formed. Concentration of nascent hydrogen, which easily dissolves in liquid metal, increases, which is often the reason for gas defects in castings [8,10,17,27].

The flash pyrolysis process of sands with selected binders corresponding to moulds pouring with liquid metals was described in the paper [28], while in this work, the results of investigations of gases emission (PAHs and BTEX) formed in foundry plants are presented—small scale chamber during pouring, cooling of moulds and knocking out of casting.

## 2. Materials and Methods

Six types of moulding sands were selected for testing: two sands bonded by binders based on synthetic resins (code phenol-formaldehyde MA and furan MF), three sands with inorganic binders (code MI, MG, MC) and one sand with bentonite (code MB) on the matrix of 100% fresh sand. Notations of individual sands are given in Table 1. The ratio of sand to metal was 2.8 to 3.0 (weight of metal was about 22 kg). Temperature of liquid cast iron was 1380–1420 °C. Tests were performed in the foundry plant HARDKOP in Trzebinia. Chemical analyses of gases were performed at the Institute of Ferrous Metallurgy in Gliwice and AGH, University of Science and Technology in Krakow.

**Figure 1:**
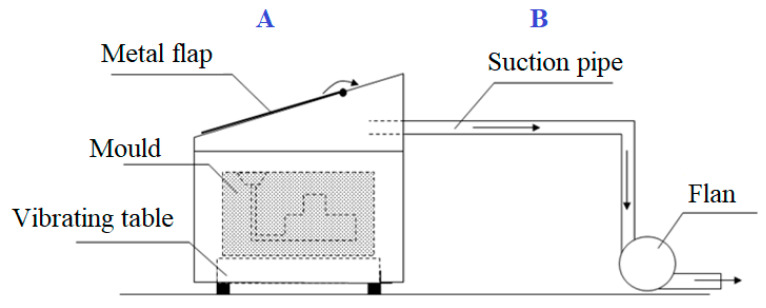
The scheme of the stand for pouring, cooling and shaking-out: (**A**) pouring stand, (**B**) suction system.

**Figure 2:**
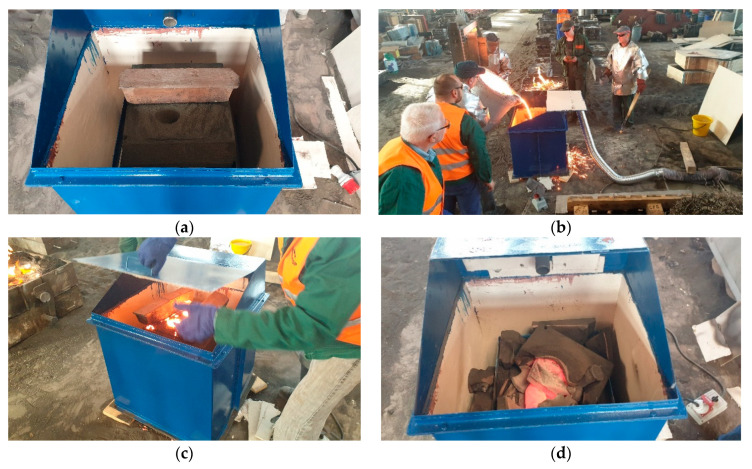
The view of the testing unit during different stages of the tests (**a**) mould sand prepared for pouring (**b**) pouring the mould sand (**c**) cooling of the mould sand (**d**) knocking out the casting.

Prepared moulds were placed on a vibrating table, the construction of which, after the pouring and cooling of the mould, allows knocking out the casting, without having to dismantle the stand. The whole system was placed in a metal box with a flap opened in the upper part, through which liquid metal was poured into the mould. The box was equipped with a connector, through which gases generated in the process were sucked. The scheme of the measuring stand is shown in Figure 1 and the view of the testing unit during different stages of the tests is shown in Figure 2.

A pipe connector was installed in the pouring stand through which gas from the stream of gases released from the mould was taken by sorption pipes at a rate of 5 L/min for PAHs and 10 L/min for BTEX, respectively. The adsorbents used in the research were: polyurethane foam and XAD resin (for adsorption of PAHs compounds) and active carbon (for adsorption of BTEX compounds). 

The composition of gases may undergo significant changes depending on the binder used for the mould preparation. For this reason, sorption tubes were designed in such a way that in the case of both low and very high concentrations of analytes in the gas stream, complete adsorption of them was possible. For this purpose, several layers of sorbents were placed in sorption tubes. This made possible to assess whether the sorbent masses used in the first layers were sufficient and, at the same time, guarantee, with their possible "breakthrough", the adsorption of analytes on subsequent layers. Sorption tubes had an internal diameter of 20 mm. In tubes used for sampling PAH compounds from the gas stream, three one-gram XAD resin layers and two layers of polyurethane foam (PUF) with a height of about 40 mm were used. In tubes used to sampling BTEX compounds from the gas stream, three one-gram layers of activated carbon separated with quartz wool were used. In addition, quartz cotton wool was used as the first layer in each sorption tube to separate the dust present in the gas. 

For the control of the analytical process, an internal control standard was added to each first layer of sorption tube just before the gas sample was taken. In the case of analysis of BTEX compounds, it was benzene d6, while for the analysis of compounds from the PAHs group, the internal standard contained: p-terphenyl d14, and 2,4,6-tribromophenol.

## 3. Determination of Compounds of Released Gasses 

### 3.1. Determination of Compounds from the PAHs and BTEX Groups

After the gas sampling process was completed, sorption sealed tubes were stored in the refrigerator until desorption of organic compounds. For desorption of PAHs contained in analytes, the XAD-2 adsorbent, PUF, and dust collected on quartz wool were transferred to an extraction thimble, which was placed in Soxhlet apparatus. For desorption of BTEX contained in analytes, the Cads adsorbent was transferred to a sintered glass column and extraction was carried out with diethyl ether. Extraction was carried out for both the first and second active carbon layers. If a signal characteristic of BTEX compounds was observed in the second layer extract after the GCMS analysis, then the extraction of the third layer compounds was continued.

### 3.2. Gas Chromatography Technique Combined with the Mass Spectrometry 

Identification and quantitative analysis of compounds from the BTEX and PAHs groups released during the process of pouring and cooling moulds and knocking out of castings were carried out using the HRGCHRMS Finnigan MAT95X (San Jose, CA, USA) system from Thermo Electron Corporation. The components of the analysed extracts were separated using a Trace GC Ultra gas chromatograph, equipped with a Supelco DB—5 ms capillary column, 30 m long, 0.25 mm internal diameter. The column can operate at temperatures from −60 °C to 325 °C. The Trace GC Ultra chromatograph enables operation within the range from an ambient temperature (practically from about 30 °C) to 350 °C. Identification of individual components leaving the chromatographic column was carried out using a Finnigan MAT95X mass spectrometer, which acts as a detector in the measuring system. 

## 4. Results and Discussion

The results of releasing substances from the PAHs group are shown in Table 2, while from the BTEX group in Table 3. They are given for the whole process (pouring, cooling and knocking out) in recalculation for 1 kg of liquid metal and 1 kg of moulding sand for six tested moulding sands on the fresh sand matrices. 

**Figure 3:**
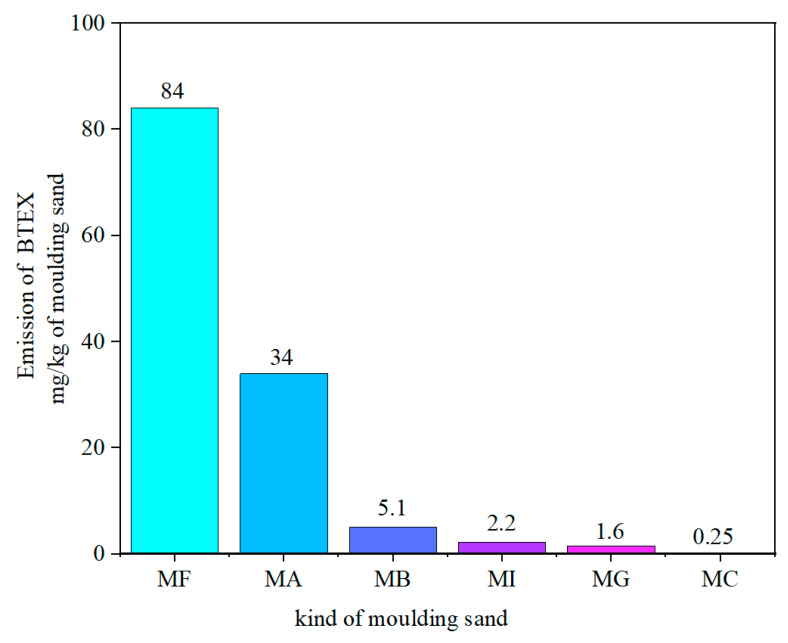
Emission of BTEX from moulding sands in industrial conditions.

The dependence of the release of compounds from the BTEX group under industrial conditions for the tested binders is shown in Figure 3.

### 4.1. Emission of Substances from the BTEX Group 

Out of two tested organic binders, a higher total emission of substances from the BTEX group indicated the MF binder (Table 3). This emission was nearly three times higher than in case of the MA binder. The main component emitted from the MA binder was benzene, while from the MF binder toluene. Moulding sand of the green sand type emitted also compounds from the BTEX group (mainly benzene and toluene) but in 10-times smaller amounts.

Tested three inorganic binders had similar, very small emissions of substances from the BTEX group. The best was the MC binder, for which the total emission of substances from the BTEX group was equal 0.25 mg/kg of moulding sand and 0.73 mg/kg of casting.

### 4.2. Emission of Substances from the PAHs Group

Emission of eight substances from the PAHs group (of the lowest boiling temperatures) was found (Table 2). The highest concentration of these substances was in gases emitted from sands with the binder based on phenol-formaldehyde resin (MA). The second sand with the organic binder (MF) indicated much lower emission of these substances in recalculation for 1 kg of moulding sand. Similar emission was measured for the sand with bentonite (MB).

When comparing emissions of substances of the PAHs group from sands with inorganic binders, a significantly higher emission is noticed from the MG sand. It is caused by the fact that binders MI and MC were hardened only by heating, while MG by organic hardener, which—in consequence—caused emitting more substances of this group.

The composition and concentration of gases emitted from moulding sands within a given group are significantly influenced by the pouring temperature, type of hardening methods and hardeners [8,15,16,18,26,29,30] were testing the temperature influence on the composition of gases emitted during the pyrolysis of the moulding sand with furan resin under semi-technical conditions (experimental mould). In order to differentiate temperatures, the mould was poured with cast iron (1400–1420 °C), bronze (1180–1200 °C) and aluminium alloy (700–720 °C). Furan resin of 50–55% of free furfuryl alcohol, free phenol < 1% and free formaldehyde 0.2–0.5% was hardened by p-toluenesulfonic acid (PTS) and benzenosulfonic (BS). The temperature influence on the amount and composition of the gases emitted from the aromatic hydrocarbons was very visible at the temperature range 700 °C–1200 °C. This specifically concerned toluene, whose emission from the mould poured by Al alloy was two times lower than from the mould poured by bronze or cast iron. For aldehydes emission, the temperature influence is smaller. However, the largest differentiation occurred in the case of CO, where emission from the mould poured with cast iron was nearly 10-times larger than from the mould poured by bronze. Probably this was mainly caused by a high carbon content in cast iron, not by the temperature (which difference was approximately 200 °C). When BS acid instead of PTS acid was applied as a hardener, the CO emission was nearly twice smaller in the case of iron casting. Moreover, from the binder hardened by PTS acid mainly toluene is emitted, while from the binder hardened by BS acid mainly benzene [30]. Investigations concerning the emission of substances from the BTEX and PAHs groups during the thermal decomposition of moulding sands with furan resin, were presented in [8,15,29,30,31].

Investigations performed at the temperature range 500–1300 °C as a flash pyrolysis, indicated that for each compound from the BTEX group it is possible to determine the temperature range at which its emission is the highest [32].

The free furfuryl alcohol content within the range: 25–72% did not have in practice any influence on the PAHs emission, which was equal app. 10–11 mg/kg of sand. For resins of a lower content of free furfuryl alcohol, naphthalene was the main compound from the PAHs group. On the other hand, the hardener influence (sulfonic acids) was noticeable. Partial substitution of p-toluenesulfonic acid (65% water solution) by lactic acid (36–41% water solution of p-toluenesulfonic acid + 30–33% water solution of lactic acid) caused the PAHs emission reduction by app. 30%. When phosphoric acid (V) was applied as a hardener, the HAPs emission was app. 3 mg/kg of sand, while it was 10 mg/kg when PTS acid was applied [3,4].

However, in the compounds of the BTEX group, neither the substitution in the hardener a part of PTS acid by lactic acid, nor free furfuryl alcohol content in resin had any influence on the emission of these compounds. This emission was at the level: 560–660 mg/kg of sand. The main, and in practice, the only, compound emitted in all experiments was benzene (the benzene fraction in the BTEX group was nearly 99%) [26].

The main objective of the assessment of the harmfulness of moulding sand is to limit their negative impact on the environment and working conditions. For this purpose, races for modification of resins and hardeners are carried out.

Investigations of the composition of gases emitted in individual operations of producing castings indicate that when the matrix of 100% of fresh sand is applied, amounts of gases emitted during the mould pouring and cooling are a few times higher than during casting knocking out. As far as the reclaim fraction in the matrix is increasing, these differences are becoming smaller and smaller. When the mould is made in 100% of the reclaim, the amount of gases evolving at the pouring and cooling stage is only 1.5-times higher than at the knocking out stage. In the whole process of casting production, naphthalene constituted 72% of gases from the PAHs group. The second in amount was phenanthene.

## 5. Conclusions

In order to compare the harmfulness of the tested moulding sands, measurements of amounts of emitted substances from the BTEX and PAHs groups under an influence of high temperatures were performed. Measurements were conducted for the whole cycle, including pouring, cooling and knocking out, within the Action B Tests in foundry plants – small scale laboratory. The obtained results were recalculated into the emission from 1 kg of the moulding sand and 1 kg of the binder applied in the given technology. 

The following conclusions can be drawn on the bases of tests performed under the small-scale chamber conditions:

1. Emissions of PAHs, as well as BTEX in case of moulding sands with organic binders, are several dozen higher than the emission of these compounds from moulding sands with inorganic binders.
2. Green sands in respect of the PAHs emission are in the intermediate sphere, while in respect of the BTEX emission are comparable with moulding sands with inorganic binders.
3. From the comparison of moulding sands with organic binders, it results that the BTEX emission from the MA sand is more than two times lower than the emission from the MF sand, while benzene and toluene predominate in the composition of gases emitted from both sands.
4. Moulding sands with inorganic binders are comparable in terms of the emission amount of substances from the BTEX and PAHs groups. Higher values of the unitary emission from moulding sands with MG binder are the result of using the organic liquid hardener for this binder hardening, while for the hardening of the remaining two binders (MI, MC) only high temperatures were used.
5. Moulding sands with inorganic binders (MG, MC and MI) are characterised by lower harmfulness for the environment and employees than moulding sands with organic binders.
6. Relatively environment friendly were green sands (MB), in which a part of coal dust was substituted by additions able to produce lustrous carbon.
7. At present, investigations concerning furan-based binders are being developed in two directions [33,34,35].
  Hardener modifications leading to:
  − The reduction of sulphur content (e.g., by the improved elimination of sulfonic acids), which will decrease SO_2_ emission, and thus will limit the harmfulness of this technology, as well as will limit the degradation of spheroidal and vermicular graphite in castings surface layers;
  − The limitation of evolving aromatic compounds amounts.
  Resin modification, which contains:
  − Limitation of the free furfuryl alcohol content to < 25 %, at maintaining comparable properties of moulding sands;
  − Increase in resin reactivity, which will allow to decrease the added hardener amounts;
  − Reduction of the formaldehyde content, to improve work conditions;
  − Reduction of the nitrogen content, even to the zero level, to eliminate gaseous defects, such as pinholes, and to limit the nitrogen oxides (NO_x_) emission.
8. In the future, silicate-based binders will have an increasing share in the technology of moulding sand due to their inorganic nature and relatively low harmfulness.
9. It would be advantageous to develop a standardized method of assessing the harmfulness of moulding sand in terms of the release of hazardous gases in the process of making castings, so that the materials used in different countries could be compared.

## Figures and Tables

**Table 1:** Types of moulding sands and their codes.

Code of the Moulding Sand	Technology of Moulding Sand
MF	Mould sand with furan resin
MA	Mould sand with phenol-formaldehyde resin
MB	Green sand
MI	Mould sand with inorganic binder
MG	Mould sand with inorganic binder
MC	Mould sand with inorganic binder

**Table 2:** Amounts of compounds from the PAHs group emitted during pouring, cooling, and knock-out (total).

Code	Naphthalene t_b_ = 217.9 °C	Acenaphthylene t_b_ = 280 °C	Acenaphthene t_b_ = 279 °C	Fluorene t_b_ = 295 °C	Phenanthene t_b_ = 340 °C	Anthracene t_b_ = 319.3 °C	Fluoranthne t_b_ = 384 °C	Pyrene t_b_ = 404 °C	Total PAHs
	(mg/kg)^a^	(mg/kg)^a^	(mg/kg)^a^	(mg/kg)^a^	(mg/kg)^a^	(mg/kg)^a^	(mg/kg)^a^	(mg/kg)^a^	(mg/kg)^a^
	(mg/kg)^b^	(mg/kg)^b^	(mg/kg)^b^	(mg/kg)^b^	(mg/kg)^b^	(mg/kg)^b^	(mg/kg)^b^	(mg/kg)^b^	(mg/kg)^b^
**MF**	0.12	0.001	0.003	0.008	0.011	0.006	0.003	-	0.15
	0.33	0.004	0.009	0.022	0.030	0.017	0.009	-	0.42
**MA**	0.51	0.031	0.012	0.032	0.031	0.015	0.006	0.005	0.64
	1.43	0.087	0.035	0.091	0.087	0.043	0.017	0.013	1.8
**MB**	0.13	0.011	-	0.006	0.012	0.006	0.003	0.005	0.16
	0.36	0.030	-	0.017	0.035	0.017	0.009	0.013	0.48
**MI**	0.015	-	-	0.001	0.003	-	-	-	0.019
	0.043	-	-	0.004	0.009	-	-	-	0.056
**MG**	0.073	0.012	0.054	0.009	0.003	0.001	0.001	0.001	0.15
	0.21	0.035	0.15	0.026	0.009	0.004	0.004	0.004	0.41
**MC**	0.009	0.001	0.001	0.003	0.001	0.001	0.001	0.001	0.018
	0.026	0.004	0.004	0.009	0.004	0.004	0.004	0.004	0.059

(mg/kg)^a^ per 1 kg of moulding sand; (mg/kg)^b^ per 1 kg of metal; t_b_ = boiling poi.

**Table 3:** Amounts of compounds from the BTEX group emitted during pouring, cooling and knock - out (total) (in recalculation for 1 kg of moulding sand and 1 kg of metal).

Code	Benzene	Toluene	Ethylbenzene	m + p -xylene	o-xylene	Total BTEX
	(mg/kg)^a^	(mg/kg)^a^	(mg/kg)^a^	(mg/kg)^a^	(mg/kg)^a^	(mg/kg)^a^
	(mg/kg)^b^	(mg/kg)^b^	(mg/kg)^b^	(mg/kg)^b^	(mg/kg)^b^	(mg/kg)^b^
**MF**	18	63	0.46	2.1	0.46	84
	52	178	1.3	6.1	1.3	238
**MA**	23	7.2	0.31	3.1	0.46	34
	65	20	0.87	8.7	1.3	96
**MB**	2.6	1.7	0.15	0.46	0.15	5.1
	7.4	4.8	0.43	1.3	0.43	14
**MI**	1.1	0.46	0.15	0.31	0.15	2.2
	3.0	1.3	0.43	0.87	0.43	6.0
**MG**	1.1	0.31	0.05	0.15	0.01	1.6
	1.0	0.87	0.13	0.43	0.04	3.5
**MC**	0.15	0.06	0.01	0.03	-	0.25
	0.43	0.17	0.04	0.09	-	0.73

(mg/kg)^a^ of moulding sand; (mg/kg)^b^ of metal.
